# *USNAP*: fast unique dense region detection and its application to lung cancer

**DOI:** 10.1093/bioinformatics/btad477

**Published:** 2023-08-01

**Authors:** Serene W H Wong, Chiara Pastrello, Max Kotlyar, Christos Faloutsos, Igor Jurisica

**Affiliations:** Osteoarthritis Research Program, Division of Orthopedic Surgery, Schroeder Arthritis Institute, and Data Science Discovery Centre for Chronic Diseases, Krembil Research Institute, University Health Network, 60 Leonard Avenue, Toronto, ON M5T 0S8, Canada; Osteoarthritis Research Program, Division of Orthopedic Surgery, Schroeder Arthritis Institute, and Data Science Discovery Centre for Chronic Diseases, Krembil Research Institute, University Health Network, 60 Leonard Avenue, Toronto, ON M5T 0S8, Canada; Osteoarthritis Research Program, Division of Orthopedic Surgery, Schroeder Arthritis Institute, and Data Science Discovery Centre for Chronic Diseases, Krembil Research Institute, University Health Network, 60 Leonard Avenue, Toronto, ON M5T 0S8, Canada; Department of Computer Science, Carnegie Mellon University, 5000 Forbes Avenue, Pittsburgh, PA 15213, United States; Osteoarthritis Research Program, Division of Orthopedic Surgery, Schroeder Arthritis Institute, and Data Science Discovery Centre for Chronic Diseases, Krembil Research Institute, University Health Network, 60 Leonard Avenue, Toronto, ON M5T 0S8, Canada; Department of Computer Science, University of Toronto, 40 St. George Street, Room 4283, Toronto, ON, M5S 2E4, Canada; Department of Medical Biophysics, University of Toronto, Princess Margaret Cancer Research Tower, MaRS Centre, 101 College Street, Room 15-701, Toronto, ON, M5G 1L7, Canada; Institute of Neuroimmunology, Slovak Academy of Sciences, vvi, Dubravská cesta 9, 845 10 Bratislava 45, Slovakia

## Abstract

**Motivation:**

Many real-world problems can be modeled as annotated graphs. Scalable graph algorithms that extract actionable information from such data are in demand since these graphs are large, varying in topology, and have diverse node/edge annotations. When these graphs change over time they create dynamic graphs, and open the possibility to find patterns across different time points. In this article, we introduce a scalable algorithm that finds unique dense regions across time points in dynamic graphs. Such algorithms have applications in many different areas, including the biological, financial, and social domains.

**Results:**

There are three important contributions to this manuscript. First, we designed a scalable algorithm, *USNAP*, to effectively identify dense subgraphs that are unique to a time stamp given a dynamic graph. Importantly, *USNAP* provides a lower bound of the density measure in each step of the greedy algorithm. Second, insights and understanding obtained from validating *USNAP* on real data show its effectiveness. While *USNAP* is domain independent, we applied it to four non-small cell lung cancer gene expression datasets. Stages in non-small cell lung cancer were modeled as dynamic graphs, and input to *USNAP*. Pathway enrichment analyses and comprehensive interpretations from literature show that *USNAP* identified biologically relevant mechanisms for different stages of cancer progression. Third, *USNAP* is scalable, and has a time complexity of $O(m+m_{c}\;\text{log}\;n_{c}+n_{c}\;\text{log}\;n_{c})$, where *m* is the number of edges, and *n* is the number of vertices in the dynamic graph; $m_{c}$ is the number of edges, and $n_{c}$ is the number of vertices in the collapsed graph.

**Availability and implementation:**

The code of *USNAP* is available at https://www.cs.utoronto.ca/∼juris/data/USNAP22.

## 1 Introduction

With a continuous expansion of disciplines where large, annotated graphs are analyzed, there is a growing need for scalable algorithms that can extract meaningful and actionable information from graph topology and node/edge annotations. Time stamps are important annotations as many real-world problems are dynamic and they evolve over time; dynamic graphs are often used to model such problems. There are different graph representations that capture different granularity of temporal information in dynamic graphs. For our purposes, a dynamic graph consists of a set of time-ordered graphs, and we refer to each graph that corresponds to a particular time stamp as a snapshot. In other words, a dynamic graph consists of a set of snapshot graphs. In this article, we propose a novel scalable algorithm such that given data modeled as a set of snapshot graphs, the algorithm finds dense regions that are specific to a given snapshot. A set of snapshot graphs with 3M edges on a Linux machine with 2.60 GHz Intel processor and 256 GB RAM took 9 s to process. The problem that we focused on in this article has broad applications in the biological, social, and business domains. For example, differentially co-expressed modules were identified when carcinogen-treated Eker rats were compared with wild-type rats (Tesson *et al.* 2010); differential co-expression gene clusters were detected in Alzheimer’s disease as well as in inflammatory and infectious diseases (Amar *et al.* 2013).

Many studies have focused on mining and analyses of dynamic graphs. Much effort has been on the tracking of changes in communities over time (e.g. Palla *et al.* 2007, Bhat and Abulaish 2015). Operations of community transformations include birth, death, growth, contraction, merge, split, continue, and resurgence (Rossetti and Cazabet 2018). Finding dense subgraphs in dynamic graphs has also received much attention. For example, Ma *et al.* (2019) detected dense temporal subgraphs. Epasto *et al.* (2015) addressed the problem of finding densest subgraphs efficiently in dynamic graphs. Galimberti *et al.* (2018) addressed the problem of finding span-cores in temporal graphs. M-zoom (Shin *et al.* 2016) finds blocks that are dense in tensors. Furthermore, community detection in dynamic networks has been much studied. For example, GraphScope (Sun *et al.* 2007) discovers communities, and determines the changing points in time of time-evolving graphs. DiTursi *et al.* (2017) identified local communities in dynamic graphs. Some researchers have summarized large dynamic graphs (e.g. Shah *et al.* 2015, Adhikari *et al.* 2017). Others have directed their attention to finding other dynamic graph mining patterns. For example, mining periodic subgraphs in temporal graphs (e.g. Qin *et al.* 2019, Zhang *et al.* 2020) has been studied in social interactions. Chan *et al.* (2008) detected spatiotemporal changes that are correlated in dynamic graphs. SpotLight (Eswaran *et al.* 2018) spots large dense subgraphs that appear or disappear suddenly. SDREGION (Wong *et al.* 2018) finds subgraphs such that their densities monotonically increase or decrease across time. Semertzidis *et al.* (2019) addressed the Best Friends Forever problem, which is to discover the most densely connected subgraphs throughout all snapshots in a graph history. They also addressed the problem of finding a subset of nodes, and a subset of *k* snapshots such that the density function over these *k* snapshots is maximized. In this article, we address a graph mining problem important for disease progression analysis, i.e. to identify dense subgraphs that are unique to a snapshot given a set of snapshot graphs. To evaluate the algorithm, we generate a dynamic graph as described in Section 4.1. Graphs representing cancer stages were input into the proposed algorithm, and dense subgraphs unique to a given tumor stage were identified.

There are different network representations that capture various levels of granularity of temporal information (Rossetti and Cazabet 2018). The static representation aggregates dynamic phenomena into a single network, and is unable to capture dynamics. The snapshot networks representation uses a sequence of time-ordered networks to model dynamic phenomena. A temporal network representation provides a fine-grain description, and a complete view of network dynamics. The choice of modeling data with snapshot networks or temporal networks will require a different design of analyses. If the data are already in network evolution states or have discretized temporal information, such as a weekly/monthly/yearly crawl of a search engine or the stages of cancer, then snapshot networks would be a natural choice. If the data have more precise temporal information, such as phone calls, emails, and short messages, then both temporal networks and snapshot networks can be used.

A class of algorithms for analyzing snapshot networks uses a two-step approach. The first step is to independently identify static subgraphs for each snapshot network, and the second step is to match the subgraphs obtained from individual snapshots. While methods for static graphs can be directly applied in this class of approaches, there are drawbacks. The major drawback is the instability of solutions from community detection algorithms (Rossetti and Cazabet 2018). It is widely acknowledged that various solutions exist for community decomposition in complex networks, and that there is not a single correct solution. The same algorithm executed on the same network except for a few topological differences may result in different solutions. In the case of stochastic algorithms, different solutions may result from the same network. Thus, it is not possible to distinguish if the changes across time are due to the network evolution or due to the instability problem. Second, the subgraph identification is based only on the information of the current time step, and has no knowledge of information from other time steps. Thus, this class of approaches may not be able to perceive more informed solutions as a result of the confined scopes. Another class of approaches for snapshot networks that circumvent the above problems is to consider all snapshots at once, and identify the desired subgraphs in a single step. In this article, we propose a novel algorithm to identify dense subgraphs that are unique to a particular snapshot using the snapshot networks representation, and consider all snapshots at once in a single step.

A few network-based approaches for identifying gene expression differences between more than two conditions have been proposed (e.g. Tesson *et al.* 2010, Ma *et al.* 2011, Amar *et al.* 2013). COSINE uses a genetic algorithm to identify a sub-network that has maximal alternation in expression patterns (Ma *et al.* 2011). DiffCoEx finds differences in gene co-expression between multiple conditions based on clustering on a dissimilarity matrix (Tesson *et al.* 2010). Hierarchical clustering was used in their analysis. DICER identifies gene sets that are differentially correlated in one class when compared with other classes using average-linkage hierarchical clustering (Amar *et al.* 2013). We propose a novel algorithm that identifies dense unique subgraphs across time points in dynamic graphs. There are major differences between these approaches and *USNAP*. First, these approaches have a different objective than *USNAP*. We designed *USNAP* to solve another dynamic graph mining problem, i.e. to identify dense subgraphs that are unique to a snapshot given a set of snapshot graphs. Second, *USNAP* is scalable and runs in quasilinear time (see Lemma 2), which is important as data continue to grow. Third, methods discussed above are designed for expression data, but *USNAP* is designed for the broader interest of complex graphs. The input to *USNAP* is a dynamic graph, thus, any data that can be represented as graphs are applicable, e.g. our algorithm can be applied to brain networks, social networks, communication networks, and financial networks. *USNAP* looks for network structural differences between different snapshots.

The scalability of *USNAP* is an important contribution. *USNAP* runs in quasilinear time, and can process a set of snapshot graphs with 3M edges on a Linux machine with 2.60 GHz Intel processor and 256 GB RAM in 9 s. *USNAP* can handle large graphs with fast response times. Importantly, *USNAP* provides a lower bound of the density measure in each step of the greedy algorithm.

*USNAP* has been designed to meet the needs of different applications. The input to the algorithm is a set of snapshot graphs. A snapshot graph is a graph with vertices and edges as defined in Section 2.1. The set can be an ordered set, e.g. when snapshots correspond to time stamps in a dynamic graph. The set can also be an unordered set, e.g. when snapshots correspond to different conditions of some clinical experiments. Furthermore, *USNAP* provides flexibility to the definition of unique subgraphs (see Section 2.4). Some applications may be natural for a unique subgraph to have all edges to be unique to a particular snapshot. Other applications may be natural for a unique subgraph to have most edges to be unique to a snapshot but allows for a fraction of edges to be shared with few other snapshots. The identified subgraphs that are unique to a given snapshot are referred to as *usnaps*.

**Figure 1.**
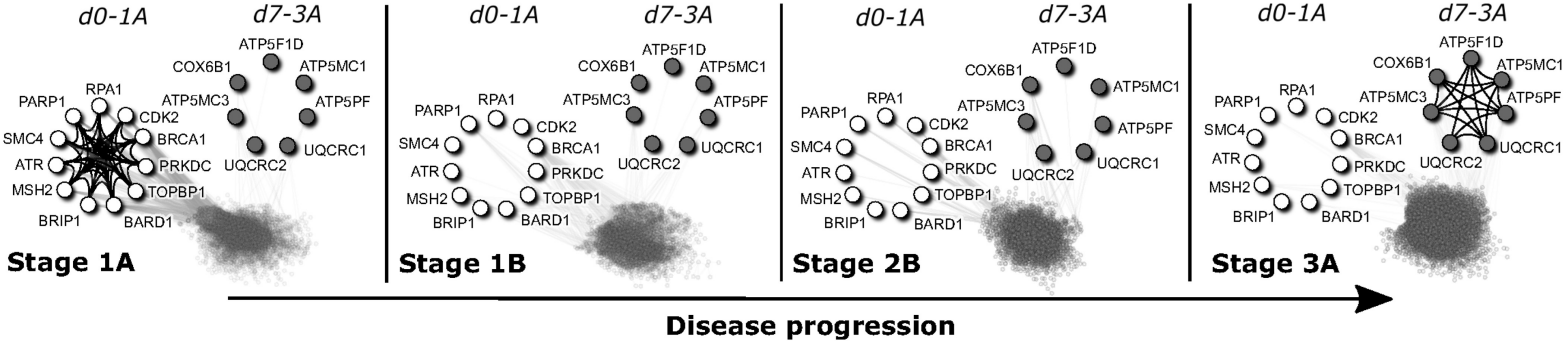
*USNAP* detects unique dense subgraphs in the *ChitaleMA1* dataset. *Usnap d0* subgraph that is specific to stage 1A (*d0 − 1A*) is shown as open circles, and *usnap d7* subgraph that is specific to stage 3A (*d7 − 3A*) is shown as closed circles. *d0 − 1A* is only present in 1A, and not in any other stages; *d7 − 3A* is only present in 3A, and not in any other stages (see the corresponding *d0 − 1A*, *d7 − 3A* in other disease stages). *d0 − 1A* is composed of DNA repair genes, and the deregulation of DNA repair functions are important to initial stages of lung cancer showing that relevant cancer mechanisms are captured in our results. *d7 − 3A* is composed of mitochondrial proteins with the majority being subunits of the ATP synthase. The increase of ATP synthase activity is important for the migration and metastasis of cancer cells.

While *USNAP* can be applied to any domain, we have applied it to four non-small cell lung cancer (NSCLC) gene expression datasets to show its effectiveness in identifying biologically relevant subgraphs across individual stages of tumor progression. The stages of tumor in each dataset were modeled as a dynamic graph, which was input into *USNAP*. Dense subgraphs unique to a given tumor stage were identified. The effectiveness is shown through the pathway enrichment analysis and detailed biological interpretation of the *usnaps*. Both the pathway enrichment analysis and the detailed study showed that *usnaps* returned by *USNAP* are highly relevant to cancer, and they capture mechanisms at different stages of tumor progression. In particular, *usnaps* capture mechanisms related to DNA repair genes, neurotransmitter receptors, voltage gated potassium channels, ATP synthase, apoptosis, and mitochondria that align with literature. Figure 1 and Supplementary Fig. S1 are *usnaps* that depict mechanisms in different stages of lung cancer. For example, two aspects of mitochondria producing energy to support the fast proliferation of cancer cells are captured in the pathway enrichment analysis and the detailed study, respectively. A detailed discussion will be presented in Section 4.

## 2 Preliminaries and problem definition

In this section, we define the problem and introduce notations that are used throughout the article. Section 2.1 states the notations, and Section 2.2 defines the collapsed graph. Section 2.3 introduces the objective function, and Section 2.4 states the exclusive threshold used in *USNAP*. Section 2.5 presents the problem definition.

### 2.1 Preliminaries

Let $G=\{G_{1},G_{2},\ldots ,G_{T}\}$ be a set of *T* snapshot graphs. Let $G_{t}(V_{t},E_{t})$, t∈[1,T] denote a graph in G where $V_{t}$ is the set of vertices, and $E_{t}$ is the set of edges in $G_{t}$. Let e(u,v,i) denote an edge with vertices *u*, *v* in snapshot *i*. Let $m=\sum_{1}^{T}\left(|E_{t}|\right)$ denote the number of edges in G, and n=|V| denote the number of vertices in G, where $V=\underset{t}{\cup }V_{t}$. Let u∈{1…T} be the specific snapshot that is desired, and $G_{u}$ be the graph that represents snapshot *u*.

### 2.2 The collapsed graph

From G, a collapsed graph, $G_{c}$, is generated. Edges in snapshots {1..T} with the same vertices (a,b), where a,b∈V will be collapsed to form an edge, $e(a,b)\in E(G_{c})$ if e(a,b,u) is an edge in $E(G_{u})$.

Let $\mathrm{NoCond}(e(a,b))=\sum_{i=1}^{T}f(e(a,b,i))$ be the number of snapshots that have an edge with vertices (a,b), where:

(1) $$f(e(a,b,i))=\{\begin{array}{cc} 1 & e(a,b,i)\in E(G_{i})\\ 0 & e(a,b,i)\notin E(G_{i})\;\text{where}\;i\in \{1..T\}. \end{array}$$

$G_{c}$ is a weighted graph with the following weight function, $e\in E(G_{c})$:

(2) $$w(e)=\{\begin{array}{cc} 2[1+\log(\frac{T}{\mathrm{NoCond}(e)})] & e\in E(G_{u})\;\mathrm{and}\;\mathrm{NoCond}(e)=1\\ 1+\log(\frac{T}{\mathrm{NoCond}(e)}) & e\in E(G_{u})\;\mathrm{and}\;\mathrm{NoCond}(e)>1 \end{array}$$

The weight function is modified from the inverse document frequency measure (Spärck Jones 1972) in the information retrieval domain.

Let $m_{c}$ be the number of edges in $G_{c}$, and $n_{c}$ be the number of vertices in $G_{c}$. Let $S_{c}$ be a subgraph in $G_{c}$. Let $E_{u}(S_{c})\subseteq E(S_{c})$ s.t. if $e\in E_{u}(S_{c})$ then $e\in E(G_{u})\;\text{and}\;e\notin E(G_{l})\mathrm{where}\;l\in \{1..T,l\neq u\}$, and *e* is called an *u* specific edge. Note that $E_{u}$ is not the same as $E(G_{u})$.

Let $d_{u}(v)$ denote the number of *u* specific edges that *v* is involved with in $G_{c}$. Let d(v) denote the degree of vertex *v* in $G_{c}$, and $d_{w}(v)$ be the weighted degree of *v* in $G_{c}$, $d_{w}(v)=\sum_{e(u,v)\in E(G_{c})}w(e)$. Let $d_{w{\left(v\right)}_{\min}}$ denote the minimum of $d_{w}(v)\forall v\in V(G_{c})$, and $d_{u_{\min}}$, $d{\left(v\right)}_{\min}$ be $d_{u}(v)$, d(v), respectively for this particular *v*. Let N(v) denote the set of neighbors of *v* in $G_{c}$. Let $\mathrm{mass}(G_{c})$ denote $\sum_{e\in E(G_{c})}w(e)$.

### 2.3 Objective function

We extend the classic density function (Charikar 2000) to weighted graphs as the objective function:

(3) $$\mathrm{density}(G_{c})=\frac{\sum_{e\in E(G_{c})}w(e)}{\left|V(G_{c})\right|}.$$

The desired property of the objective function is to return dense subgraphs such that if two subgraphs have the same number of nodes, the subgraph with the higher mass is denser than the other. Formally, let $S_{c},S_{c}^{\prime }$ be two subgraphs in $G_{c}$ such that $\left|V(S_{c})|=|V(S_{c}^{\prime })\right|$ and $\mathrm{mass}(S_{c})\geq \mathrm{mass}(S_{c}^{\prime })$, then $\mathrm{density}(S_{c})\geq \mathrm{density}(S_{c}^{\prime })$. This objective function satisfies the desired property. Recall that weights in the edges of $G_{c}$ reflect the importance of the edges in terms of their uniqueness.

### 2.4 Exclusive threshold

The subgraphs returned have to maximize the objective function, and have to satisfy the exclusive threshold. The exclusive threshold is that the fraction of edges in the subgraph specific to *u* has to be at least a predefined threshold from the input parameter. Formally, the exclusive threshold is:

(4) $$\frac{\left|E_{u}(S_{c})\right|}{\left|E(S_{c})\right|}\geq \gamma \in (0,1],$$

where $S_{c}$ is a subgraph in $G_{c}$.

### 2.5 Problem definition

Given a set of snapshot graphs, G, the desired unique snapshot, *u*, the number of subgraphs, *k*, find *k* subgraphs that

1. maximize the objective function, density
2. the exclusive threshold is satisfied.

## 3 Materials and methods

### 3.1 Algorithm

*USNAP* is a heuristic algorithm, since naive enumeration of all possible subgraphs is combinatorial, which is not scalable.

*USNAP* first generates a collapsed graph, $G_{c}$, from G, a set of *T* snapshot graphs. *USNAP* then starts with the entire collapsed graph, and finds one *usnap* in each iteration. *USNAP* then removes the discovered *usnap* from $G_{c}$, and continues to search for another *usnap*. Pseudo code and design details of *USNAP* are in the Supplementary Material.

The weights of the edges of the collapsed graph reflect the importance (uniqueness) of the edges. Intuitively, higher weight edges mean that fewer snapshots have these edges while lower weight edges mean that more snapshots have these edges. *u* specific edges will have the highest weight. Since the goal of *USNAP* is to find dense subgraphs that are specific to a snapshot, the weight function in the collapsed graph is designed so that *USNAP* is biased toward picking edges with unique or few snapshots.

*USNAP* begins with $G_{c}$, and greedily searches for unique dense regions. *USNAP* removes a vertex at a time, greedily selecting a vertex according to Lemma 1. *USNAP* then returns a graph configuration that has the maximum density value, and satisfies the exclusive threshold.

Lemma 1. The removal of  $v\in V(G_{c})$ such that $d_{w}(v)(1+\frac{d_{u}(v)}{d(v)})$ is minimized results in

$$\frac{\mathrm{mass}(G_{c})-d_{w}{\left(v\right)}_{\min}}{|V|-1}\geq \mathrm{density}(G_{c}^{\prime })\geq \frac{\mathrm{mass}(G_{c})-2d_{w}{\left(v\right)}_{\min}}{|V|-1},$$

where $G_{c}^{\prime }$ is a collapsed graph such that $V(G_{c}^{\prime })=V(G_{c})\setminus \{v\}$.

More information and the proof to Lemma 1 can be found in the Supplementary Material. Our goal is to maximize density while satisfying the exclusive threshold. The optimal value for $\mathrm{density}(G_{c}^{\prime })$ would be to subtract $d_{w}{\left(v\right)}_{\min}$ from $\mathrm{mass}(G_{c})$ in the numerator resulting in $\mathrm{density}(G_{c}^{\prime })=\frac{\mathrm{mass}(G_{c})-d_{w}{\left(v\right)}_{\min}}{|V|-1}$. Lemma 1 proves that in each step of the greedy algorithm, $\mathrm{density}(G_{c}^{\prime })$ could attain to the optimal value and would not be worse than $\frac{\mathrm{mass}(G_{c})-2d_{w}{\left(v\right)}_{\min}}{|V|-1}$. Note that the lower bound is to subtract two times $d_{w}{\left(v\right)}_{\min}$ from $\mathrm{mass}(G_{c})$ while the optimal value would be to subtract $d_{w}{\left(v\right)}_{\min}$ from $\mathrm{mass}(G_{c})$ in the numerator. The function that is used to select which *v* to remove in $V(G_{c})$ in Lemma 1 has two aspects: $d_{w}(v)$ is to have dense subgraphs, and $\left(1+\frac{d_{u}(v)}{d(v)}\right)$ is to discourage the removal of nodes that have *u* specific edge(s). The more *u* specific edges *v* has, the greater $\left(1+\frac{d_{u}(v)}{d(v)}\right)$ will be.

### 3.2 Time complexity and scalability

Lemma 2. *USNAP* has a time complexity of $O(m+m_{c}\;\text{log}\;n_{c}+n_{c}\;\text{log}\;n_{c})$.

Recall that *m* is the number of edges in G, and *n* is the number of vertices in G. $m_{c}$ is the number of edges in $G_{c}$, and $n_{c}$ is the number of vertices in $G_{c}$. The proof of Lemma 2 can be found in the Supplementary Material.

Since $m_{c}\leq m$ and $n_{c}\leq n$, the time complexity of *USNAP* in terms of the number of edges and number of vertices in the input set of *T* snapshot graphs is O(mlogn+nlogn). Expressing the time complexity with $m_{c}$, $n_{c}$ provides a tighter bound for *USNAP*.

**Figure 2.**
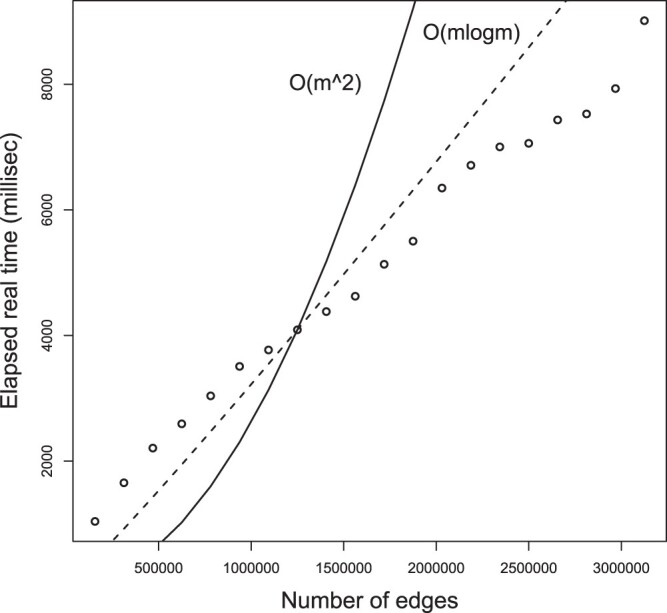
The runtime of *USNAP*. The actual runtimes are shown as black circles, $O(m^{2})$ is shown as the solid line, and O(mlogm) is shown as the dashed line. The important point to note is the comparison of different complexity functions in relation to the input size, and not the actual seconds that a given input size took. This is because the number of seconds for a given input size will change from machine to machine.

*USNAP* is designed to be quasilinear time O(mlogn). Figure 2 shows the actual running time for *USNAP* to return five *usnaps* with no restart, no fraction of vertices to remain and the exclusive threshold is one. The different sizes of graphs were generated by down-scaling the correlation graphs of the chitaleMA1 dataset. *USNAP* was implemented in Java. A machine with 256 GB RAM, and Intel(R) Xeon(R) CPU E5-2660 v3 @ 2.60 GHz, x86_64, CentOS Linux 7 (Core) was used for all experiments.

## 4 *USNAP* validation on NSCLC datasets

While *USNAP* is generic, and can be applied in any domain, we applied it to gene expression data to model different stages in tumor progression. Four NSCLC microarray gene expression datasets, referred to as *ChitaleMA1*, *ChitaleMA2*, *Okayama*, and *Raponi*, were used to demonstrate the effectiveness of *USNAP*. Refer to the Supplementary Material for more information regarding the data used.

Section 4.1 describes the input to *USNAP*. Section 4.2 presents results returned by *USNAP* with exclusive threshold equals one, with no restart, and no fraction of vertices to remain. We used a detailed study (Section 4.3) as well as pathway enrichment analyses (Section 4.4) to show that the identified *usnaps* are biologically meaningful, and are relevant to the different stages in the progression of cancer.

### 4.1 Input to *USNAP*

The input dynamic graph has *T* snapshots that correspond to the *T* stages of NSCLC in a dataset. Dynamic graphs were constructed by annotating physical protein interaction network for each individual stage, and considering only highly correlated genes overlapping with interactions. In this article, we assumed that the dynamic graphs have been generated, and are taken to be the input to *USNAP*. Refer to Supplementary Section S2 for more information on the input dynamic graphs and their construction. The input dynamic graphs are also available at https://www.cs.utoronto.ca/∼juris/data/USNAP22.

### 4.2 *USNAP*’s results

Supplementary Tables S2–S5 show the top 10 (or less if there were less results) densest *usnaps* having more than two nodes that are specific to Stages 1A, 1B, 2 or 2B, and 3A for each dataset, respectively. The tables are in sorted order of density with *d*0 being the densest *usnap* for each stage and for each dataset. All results are available at https://www.cs.utoronto.ca/∼juris/data/USNAP22.

Importantly, for all *usnaps* from all four datasets, the edges that are specific to a stage do not appear in any other stages in the same dataset. For example, *usnap d*0 in *Raponi* has a clique with 15 edges that are specific to Stage 2B (depicted in closed circles in Supplementary Fig. S1). Out of these 15 edges, no edge is present in Stage 1A, 1B or 3A of *Raponi*.

Figure 1 depicts *usnaps* that are specific to Stages 1A and 3A in *chitaleMA1*. *Usnap d*0 that is specific to 1A (in open circles) is densely connected within the *usnap*. Notice that none of these edges are present in Stages 1B, 2B, or 3A. *Usnap d*7 that is specific to 3A (in closed circles) is also densely connected within the *usnap* in Stage 3A. Once again, none of these edges are present in Stages 1A, 1B, or 2B.

### 4.3 Detailed analysis and interpretation of *usnaps*

We performed a comprehensive interpretation and analysis on *usnaps*. *USNAP* captured different mechanisms for different stages of cancer, validating *usnaps*’ meaningfulness to tumor progression.

#### 4.3.1 Observation for Stage 1A: captured risk factor that is implicated for lung cancer

*Usnap d*0 specific to Stage 1A from the Raponi dataset (Supplementary Fig. S1) is composed of genes implicated in asthma. TAS2Rs are bitter taste receptors expressed in the human bronchus, airway epithelial cells and lung macrophages (Grassin-Delyle *et al.* 2019). TAS2Rs have been implicated in airway defense mechanisms and are found to be elevated in patients with asthma. Serotonin and its receptors (e.g. HTR5A) have been implicated in the pathophysiology of asthma, and have been shown to regulate cytokine release in airway epithelial cells (Bayer *et al.* 2007). Polymorphisms in SLC6A7 have been linked to asthma (Kim *et al.* 2010), MLLT1 has been shown to be hypermethylated in allergic asthma (Cardenas *et al.* 2019) and GPR31 in non-atopic asthma (Kim *et al.* 2013). Asthma has been implicated as a risk factor for lung cancer, but mainly for the squamous cell carcinoma histology (Rosenberger *et al.* 2012). Interestingly, *d*0 is from the Raponi dataset, the only dataset among the ones analyzed that is squamous cell carcinoma data.

#### 4.3.2 Observation for Stage 1A: DNA repair function affected

*Usnap d*0 specific to Stage 1A from the ChitaleMA1 dataset is composed of DNA repair genes (Fig. 1). Most belong to the double strand break repair systems (BRIP1, BRCA1, RPA1, and BARD1) or are responsible for signaling DNA damage (ATR and TOPBP1), while the remaining belong to other DNA repair systems (mismatch repair, non-homologous end joining, and single strand break repair) (Chatterjee and Walker 2017). DNA repair is particularly important in the lungs, as their tissue is continuously exposed to a variety of insults that can induce DNA damage. DNA repair genes lead to repair of DNA and are fundamental for cell cycle progression. When such genes are mutated, the DNA can accumulate mutations and start the carcinogenic process (Mamdani and Jalal 2016). It is then obvious to find DNA repair function deregulated starting from the initial stages of lung cancer.

#### 4.3.3 Observation for Stage IB: neurotransmitter receptors are highly active in early stages of tumor progression

*Usnap d*0 (GABRG3 GABRD GABRR2 HTR3A CHRNA10 GLRA3 CHRNA6 CHRND HTR3B) from the ChitaleMA1 dataset identified by *USNAP* to be specific to Stage 1B is composed of neurotransmitter receptors (GABA—GABRG3, GABRD, GABRR2; serotonin—HTR3A/B; cholinergic—CHRNA10, CHRNA6, CHRND; and glycine—GLRA3). The neurotransmitter acetylcholine (ACh) acts as an autocrine growth factor for human lung cancer. ACh receptors mediate the cancer growth effect on lung of nicotine, the addictive component of cigarette smoke. It has been shown that ACh receptors are highly expressed in early stages of carcinogenesis and as the tumor progresses to more advanced stages the expression decreases, leaving other pathways to control tumor growth (Friedman *et al.* 2019).

Furthermore, GABA’s most known function is as a neurotransmitter in the brain, but it has been shown to be expressed and involved in the development of other tissues. GABA receptors have been shown to be highly expressed in early stages of NSCLC, but not in advanced stages, and a reduced expression of such receptors leads to worse survival (Zhang *et al.* 2013).

#### 4.3.4 Observation for Stage 1B: voltage gated potassium channel linked to the initial stages of tumor growth and proliferation

*Usnap d*1 (GRIA2 KCNC1 KCNF1 KCNS1 KCNJ14 KCNH6 KCNQ4 CNGB3) that is specific to Stage 1B from the ChitaleMA1 dataset, with 8 nodes and 24 edges, is composed mostly of potassium channels. Potassium channels are pore-forming transmembrane proteins that let potassium flow through the membrane. They are involved not only in the obvious control of the membrane potential and cell excitability, but also in the regulation of cell proliferation, migration, and apoptosis. Potassium channels are expressed in many healthy and cancer cells, and in the latter they are involved in cancerogenesis as well as metastasis formation (Comes *et al.* 2015). Potassium channels have been shown to be potential biomarkers in lung cancer and their overexpression has been linked to a lower survival rate. Interestingly, voltage gated potassium channels, the ones present in this *usnap*, have been linked to the initial stages of tumor growth and proliferation (Bulk *et al.* 2021).

#### 4.3.5 Observation for Stage 2B: embryonal development genes that linked to lung cancer progression

*Usnap d*0 (RORA HOXA2 PAX6 LEF1 HOXA11 HOXB1 LHX1 MSX2 BMP4 EVX1 POU2F1 EN2) from the ChitaleMA1 dataset that is specific to Stage 2B is composed of transcription factors linked to embryonal development. The majority belong to the homeobox gene family (HOX), a set of genes that regulate growth and organogenesis. HOX genes are expressed in healthy human lungs and play a crucial role in their development. It has been shown that many HOX genes are expressed and involved in lung carcinogenesis, and that a different set of HOX genes is activated compared to normal lung, but the number of expressed HOX genes decreases with increase of malignancy, suggesting that progression of lung cancer is linked to loss of HOX gene expression (Kappen 1996).

#### 4.3.6 Observation for Stage 3A: ATP synthase involves cancer metastasis in later cancer stages

*Usnap d*7 from the ChitaleMA1 dataset specific to Stage 3A is composed of mitochondrial proteins (Fig. 1). The majority are subunits of the ATP synthase. Metabolism is notably altered in cancer cells, and metabolic reprogramming leads to increase in glucose uptake and glycolysis, and consequent generation of ATP and lactic acid in the cytosol. Mitochondria need to produce energy through oxidative phosphorylation, regulated by mitochondrial ATP synthase, to support the fast proliferation of cancer cells. It has been shown that the inhibition of ATP synthesis can slow cancer proliferation. In later cancer stages, ATP synthase is involved in cancer progression and metastasis. In fact, it has been shown that oxidative phosphorylation increases in migrating cancer cells, compared to cells from the primary tumor (Galber *et al.* 2020). The presence of this *usnap* at stage 3A suggests that the interactions among ATP synthase subunits are being identified because of the increase of ATP synthase activity to favor migration and metastasis of the cancer cells.

### 4.4 Pathway enrichment analysis of *usnaps*

In addition to the detailed study in Section 4.3, pathway enrichment was used to evaluate the biological meaning of the results from *USNAP*.

Literature curated pathways from pathDIP (Rahmati *et al.* 2020) version 4 were used. The pathway enrichment analysis was performed using hypergeometric tests for each pathway and for each unioned *usnap*. *Usnaps* for each dataset and for each stage were unioned. The universes used for the hypergeometric tests were the genes in the chipset for each dataset. *P*-values were adjusted for multiple testing using the false discovery rate with a threshold of 0.05.

Our findings show that *usnaps* capture meaningful and stage-specific biological functions in the progression of lung cancer. Some biological functions are over-represented in early stages of cancer, while others are over-represented in later stages.

Pathways specific to a given stage, and those present in two datasets at the given stage are presented in Table 1. Each *P*-value in Table 1 is the largest *P*-value among the datasets. Table 1 displays the Top 6 (or less when fewer enrichments are available) specific pathways ranked according to the largest *P*-value. Refer to Supplementary Tables S6–S9 for the full lists of specific pathways.

**Table 1.** Pathway specific to a given stage.

Stage	Pathway	*P*-value
1A	WikiPathways-dna mismatch repair	.0014
1A	REACTOME-apoptosis	.0020
1A	BioCarta-the prc2 complex sets long-term gene silencing through modification of histone tails	.0042
1A	WikiPathways-the effect of progerin on the involved genes in hutchinson-gilford progeria syndrome	.0066
1A	REACTOME-mitotic metaphase/anaphase transition	.0143
1A	BioCarta-caspase cascade in apoptosis	.0188
1B	BioCarta-hiv-1 defeats host-mediated resistance by cem15	.0082
1B	REACTOME-interactions of rev with host cellular proteins	.0132
1B	REACTOME-early phase of hiv life cycle	.0491
2or2B	IPAVS-gp130-jak-stat	4.45E-05
2or2B	KEGG-th1 and th2 cell differentiation	5.27E-05
2or2B	INOH-cd4 *t* cell receptor signaling-erk cascade	8.16E-05
2or2B	WikiPathways-*t*-cell receptor and co-stimulatory signaling	.0001
2or2B	REACTOME-pi5p, pp2a and ier3 regulate pi3k/akt signaling	.0010
2or2B	REACTOME-regulation of kit signaling	.0013
3A	WikiPathways-parkin-ubiquitin proteasomal system	.0191
3A	WikiPathways-electron transport chain (oxphos system in mitochondria)	.0496

Stage 1A has apoptosis-specific pathways. Apoptosis is one of the pathways that is found altered in early stages, both in NSCLC and small cell lung cancer, and is linked to worse prognosis (Lu *et al.* 2020).

In Stage 1B, there is an overlap between the lung cancer signal and HIV-1. It is known that lung cancer is the most frequent malignancy non-related to AIDS in patients with HIV. It has been shown that HIV-1 Nef protein modifies fibroblasts and normal epithelial cells to activate the initial stages of lung cancerogenesis (Santerre *et al.* 2019).

In Stage 3A, parkin-ubiquitin proteasomal system is enriched. Parkin has been shown to be highly expressed in lung cancer, and its expression increases with tumor progression. Parkin binds and degrades p21 preventing its function as inhibitor of cell cycle progression (Park *et al.* 2019).

Mitochondria need to produce energy through oxidative phosphorylation, regulated by the mitochondrial ATP synthase, to support the fast proliferation of cancer cells. *USNAP* is able to capture this mechanism in Stage 3A in both the detailed analysis (Section 4.3) as well as the pathway enrichment analysis. First, in Section 4.3, we presented *usnap d*7 from the ChitaleMA1 dataset that is specific to stage 3A (Fig. 1). The majority of this *usnap* is composed of subunits of the ATP synthase. We mentioned in Section 4.3 that the presence of this *usnap* at Stage 3A suggests that the identification of interactions among ATP synthase subunits are due to the increase of ATP synthase activity to favor migration and metastasis of the cancer cells. Second, in the pathway enrichment analysis, the oxidative phosphorylation pathway is one of the pathways enriched that is specific to Stage 3A. It has been shown that oxidative phosphorylation increases in migrating cancer cells when compared to cells from the primary tumor (Galber *et al.* 2020).

## 5 Conclusions

There are three main contributions to this manuscript. First, we designed a novel algorithm, *USNAP*, that is scalable and effective in identifying dense subgraphs that are unique to a snapshot given a set of *T* snapshot graphs. Importantly, *USNAP* provides a lower bound of the density measure in each step of the greedy algorithm. Second, *USNAP* is effective in real data where insights and understanding were obtained. While *USNAP* can be applied to any domain, we applied it to four NSCLC datasets and found meaningful results. Third, *USNAP* is scalable, and has a time complexity of $O(m+m_{c}\;\text{log}\;n_{c}+n_{c}\;\text{log}\;n_{c})$ where *m* is the number of edges, and *n* is the number of vertices in the set of *T* snapshot graphs; $m_{c}$ is the number of edges, and $n_{c}$ is the number of vertices in the collapsed graph. A re-formulation of our proposed problem into a convex optimization problem could be a beneficial future contribution.

In this article, we have applied *USNAP* to lung cancer datasets to show its effectiveness in different stages of tumor progression. Stages of tumor were modeled using dynamic graphs. A detailed study as well as pathway enrichment analyses were used to show that *usnaps* returned by *USNAP* are informative, as they capture mechanisms at different stages of tumor progression. In particular, *usnaps* capture mechanisms related to DNA repair genes, neurotransmitter receptors, voltage gated potassium channels, ATP synthase, apoptosis, and mitochondria that align with literature.

## Supplementary Material

btad477_Supplementary_Data
