# Correction to: *USNAP*: fast unique dense region detection and its application to lung cancer

**DOI:** 10.1093/bioinformatics/btad736

**Published:** 2024-03-27

This is a correction to: Serene W H Wong, Chiara Pastrello, Max Kotlyar, Christos Faloutsos, Igor Jurisica, *USNAP*: fast unique dense region detection and its application to lung cancer, *Bioinformatics*, Volume 39, Issue 8, August 2023, btad477, https://doi.org/10.1093/bioinformatics/btad477

In the originally published version of this manuscript, there were errors in the affiliations list. The list should read:

“^[…]^

^2^Department of Computer Science, Carnegie Mellon University, 5000 Forbes Avenue, Pittsburgh, PA 15213, United States

^3^Department of Computer Science, University of Toronto, 40 St. George Street, Room 4283, Toronto, ON, M5S 2E4, Canada

^4^Department of Medical Biophysics, University of Toronto, Princess Margaret Cancer Research Tower, MaRS Centre, 101 College Street, Room 15-701, Toronto, ON, M5G 1L7, Canada

^5^Institute of Neuroimmunology, Slovak Academy of Sciences, vvi, Dubravská cesta 9, 845 10 Bratislava 45, Slovakia” instead of:

“^[…]^

^2^Department of Computer Science, GHC 7003 Carnegie, Mellon University, 5000 Forbes Avenue, Pittsburgh, PA 15213-3891, United States

^3^Departments of Medical Biophysics and Computer Science, University of Toronto, Toronto, ON, Canada

^4^Institute of Neuroimmunology, Slovak Academy of Sciences, Bratislava, Slovakia”.

Enumeration in the author byline has been correspondingly emended to read: “[…]Igor Jurisica^1,3,4,5,^*” instead of “[…]Igor Jurisica^1,3,4,^*”.

A reference date was erroneous and should read: “Rossetti and Cazabet 2018” instead of: “Rossetti and Cazabet 2019”.

In **1 Introduction**, second paragraph, within the 18th sentence, double quotation marks were introduced. “[…]“Best Friends Forever” problem[…]” should read “[...]Best Friends Forever problem[…]”; third paragraph, beginning of second sentence should read: “The static representation[…]” instead of: “The “static” representation[...]”; beginning of fourth sentence should read: “The snapshot networks representation[…]” instead of: “The “snapshot networks” representation[…]"; beginning of the fifth sentence should read: “A temporal network representation[…]” instead of: “A “temporal network” representation[…]”.

In section **2.2 The collapsed graph**, an errored fullstop was removed from weight function (2). The reference: “(Jones 1972)” was emended to read instead: “(Spärck Jones 1972)”.

Figure 2 was missing the line of circles representing actual runtimes. Figure 2 should read:

**Figure 2.**
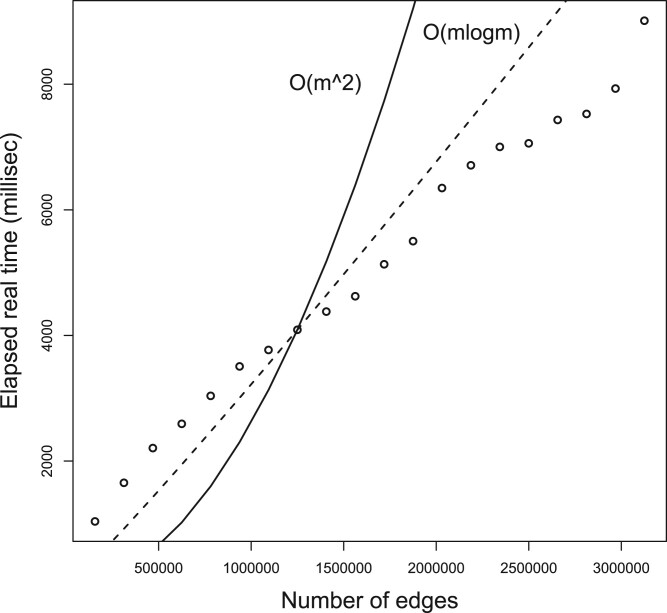
The runtime of *USNAP*. The actual runtimes are shown as black circles, $O(m^{2})$ is shown as the solid line, and O(mlogm) is shown as the dashed line. The important point to note is the comparison of different complexity functions in relation to the input size, and not the actual seconds that a given input size took. This is because the number of seconds for a given input size will change from machine to machine.

instead of:

**Figure 2.**
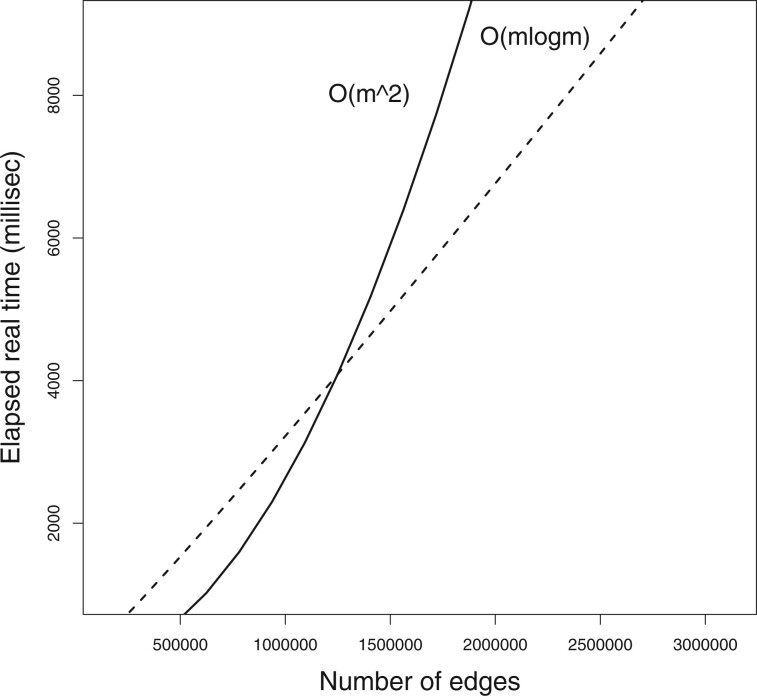
The runtime of *USNAP*. The actual runtimes are shown as black circles, $O(m^{2})$ is shown as the solid line, and O(mlogm) is shown as the dashed line. The important point to note is the comparison of different complexity functions in relation to the input size, and not the actual seconds that a given input size took. This is because the number of seconds for a given input size will change from machine to machine.

In **Section 3, 3.1 Algorithm**, fifth paragraph, within the 2nd sentence, double quotation marks were introduced. “Our goal is to maximize “density”[…]” should read: “Our goal is to maximize density[…]”. A reference date was erroneous in section **4.3.4** and should read: “(Bulk *et al.* 2021)” instead of: “(Bulk *et al.* 2020)”.

Page numbers were incomplete in the reference “Bhat SY, Abulaish M. HOCTracker: tracking the evolution of hierarchical and overlapping communities in dynamic social networks. IEEE Trans Knowl Data Eng 2015;27:1019” and should read: “Bhat SY, Abulaish M. HOCTracker: tracking the evolution of hierarchical and overlapping communities in dynamic social networks. IEEE Trans Knowl Data Eng 2015;27:1019–1031”.

There was a name error in a reference – this should read: “Spärck Jones, K. A statistical interpretation of term specificity and its application in retrieval. *J. Doc* 1972;28:11–21.” Instead of “Jones KS. A statistical interpretation of term specificity and its application in retrieval. J Doc 1972;28:11–21”. This reference was also moved to be ordered alphabetically within the list.

Reference “Rossetti G, Cazabet R. Community discovery in dynamic networks: a survey. ACM Comput Surv 2018;51:1–37.” should read instead: “Rossetti G, Cazabet R. Community discovery in dynamic networks: a survey. ACM Comput Surv (CSUR) 2018;51:1–37.”

There was an numerical error in the final characters of the last entry in the reference list. This should read: “Zhang X, Zhang R, Zheng Y *et al.* Expression of gamma-aminobutyric acid receptors on neoplastic growth and prediction of prognosis in non-small cell lung cancer. *J Transl Med* 2013;11:102” instead of: “Zhang X, Zhang R, Zheng Y *et al*. Expression of gamma-aminobutyric acid receptors on neoplastic growth and prediction of prognosis in non-small cell lung cancer. *J Transl Med* 2013;11:102–10”.

The publisher apologises for all these errors, which were introduced during the production process. These have been emended where the publication record would be seriously affected by the academic accuracy of the published information.

