# Supplementary material for: USNAP: fast unique dense region detection and its application to lung cancer
Source: Bioinformatics. 2023 Aug 1;39(8):btad477. doi: 10.1093/bioinformatics/btad477 (PMC10425186; doi:10.1093/bioinformatics/btad477)
Supplement: btad477_Supplementary_Data [file btad477_supplementary_data.pdf]

USNAP: Fast unique dense region detection and its application to lung cancer

Serene W.H. Wong, Chiara Pastrello, Max Kotlyar,
Christos Faloutsos, Igor Jurisica

1 Additional notes to the method

1.1 *USNAP*

Algorithm 1: *USNAP*

Input: A set of T snapshot graphs:$\mathcal{G}$, no. of *usnap*:k, no. of restart:k', fraction of vertices to remain:p, specific snapshot:u, exclusive threshold γ

Output: $\leq k$ *usnaps*

1 ResultSet = $\emptyset$;
2 $G_c \leftarrow$ CreateCollapsedGraph $(\mathcal{G}, u)$;
3 **while** *No. of usnaps found are* $< k$ **do**
4   *result* $\leftarrow$ FindARegion (G_c, γ);
5   **if** *No usnap is found and restart* $< k'$ **then**
6         $V(G_c) \leftarrow V(G_c) \setminus \{v\}$ where $v \in V(G_c)$ is an arbitrary vertex;
7   **else**
8   ResultSet $\leftarrow$ ResultSet $\cup \{result\}$;
9         $V(G_c) \leftarrow V(G_c) \setminus U$ where $v \in U$ are arbitrary vertices in *result* and $|U| = (1-p)*$ #vertex in *result*
10 **end**
11 **return** *ResultSet*;

Algorithm 1 gives a top level description of *USNAP*. *USNAP* first creates a collapsed graph, G_c. *USNAP* then starts with the entire collapsed graph, and finds one *usnap* in each iteration. *USNAP* then removes the discovered *usnap* from G_c, and continues to search for another *usnap*.

Note that a subgraph that satisfies the exclusive threshold may not exist for any given graph. Furthermore, it is possible that *USNAP* is not able to find any *usnap* given its greedy nature. Thus, a restart option is provided in lines 5-6. Restarts allow for possible different orderings in the removal of vertices which may lead to finding *usnaps*. The removal of a vertex will affect its neighbors, which in turn may affect which vertex is to be removed first.

To increase applicability of *USNAP*, we provide a few tunable parameters. Some applications may desire the overlapping of vertices between *usnaps* while others may desire not to have any overlaps. Line 9 provides an option for the overlapping of vertices between *usnaps*. p specifies the fraction of vertices in the identified *usnap* that should remain for the searching of other *usnaps*.

Algorithm 2: CreateCollapseGraph $(\mathcal{G}, u)$

Input: A set of T snapshot graphs:$\mathcal{G}$, specific snapshot:u
Output: Collapsed graph G_c
1 hash $edgeH$, $edgeUniqueH$;
2 **foreach** $e(a, b, z) \in E(\mathcal{G})$ **do**
3   create $edge(a, b)$;
4   put $edge(a, b)$ in $edgeH$ and increment its count;
5   **if** $z == u$ **then**
6   put $edge(a, b)$ in $edgeUniqueH$;
7 **end**
8 $E(G_c) = \emptyset$, $V(G_c) = \emptyset$;
9 **foreach** $edge(a, b) \in edgeUniqueH$ **do**
10     $V(G_c) \leftarrow V(G_c) \cup \{a, b\}$;
11     $E(G_c) \leftarrow E(G_c) \cup edge(a, b)$;
12   compute $w(edge(a, b))$ using $edgeH$;
13 **end**
14 **return** G_c

Algorithm 2 creates a collapsed graph, G_c, from $\mathcal{G}$. Hash *edgeH* contains an edge as key, and the count of the number of snapshots having a given edge as value. *edgeUniqueH* keeps track of the edges in G_u. The weight in line 12 is computed using the weight function specified in the manuscript.

Algorithm 3: FindARegion (G_c, γ)

Input: Current graph G_c
Output: 0 or 1 *usnap*
Configuration $= \emptyset$;
initialize min-heap MH;
while $|V(G_c)| \neq \emptyset$ **do**
    $v \leftarrow$ RemoveNode (G_c, MH);
    Calculate density for G_c;
    if G_c *satisfies the exclusive threshold* γ **then**
        Configuration $\leftarrow$ Configuration $\cup \{v\}$;
end
return *Configuration with the max density*;

Algorithm 3 finds one *usnap*. MH is a min-heap that stores $d_w(v)(1 + \frac{d_u(v)}{d(v)})$ for each vertex in G_c. Only the removed vertices, maximum density, and indices

for configurations are stored. Note again that not all graphs will have a *usnap*. Moreover, it is possible that no subgraph that satisfies the exclusive threshold can be found due to the greedy nature of *USNAP*.

Algorithm 4: RemoveNode (G_c, MH)

Input: Current graph G_c, min-heap MH
Output: $v \in V(G_c)$ that is removed, according to Lemma 1

1 $v \leftarrow v \in V(G_c)$ with the min value in MH;
2 $V(G_c) \leftarrow V(G_c) \setminus \{v\}$;
3 **foreach** $u \in N(v)$ **do**
4 | update u in MH;
5 **end**
6 **return** v;

Algorithm 4 removes a vertex according to Lemma 1, and performs the necessary updates. Line 1 performs the delete minimum operation in MH. It is important that the computation of the value to be updated for u be in constant time in order for line 4 to execute in $O(logn_c)$.

1.2 Lemmas

Lemma 1. *The removal of* $v \in V(G_c)$ *such that* $d_w(v)(1+\frac{d_u(v)}{d(v)})$ *is minimized results in*

$$\frac{mass(G_c) - d_w(v)_{min}}{|V|-1} \geq density(G_c') \geq \frac{mass(G_c) - 2d_w(v)_{min}}{|V|-1}$$

where G_c' *is a collapsed graph such that* $V(G_c') = V(G_c) \setminus \{v\}$.

Proof. Let $v \in V(G_c)$, and $d_w(v) = d_w(v)_{min}$. We consider the following 2 cases: $\frac{d_u(v)_{min}}{d(v)_{min}} = 0$ and $\frac{d_u(v)_{min}}{d(v)_{min}} > 0$. Note that $\frac{d_u(v)}{d(v)} \in [0,1]$, and $d(v)$ cannot be 0 as there is no vertex with no edge in G_c. The first case corresponds to when vertex v has the minimum weighted degree in G_c and it doesn't have any u specific edge. The second case corresponds to when v has the minimum weighted degree in G_c and it does have u specific edge(s).

If $\frac{d_u(v)_{min}}{d(v)_{min}} = 0$, then *USNAP* will pick v to be removed as:

$$d_w(v)_{min}(1 + \frac{d_u(v)_{min}}{d(v)_{min}}) = d_w(v)_{min}.$$

In this case, the removal of v results in

$$density(G_c') = \frac{mass(G_c) - d_w(v)_{min}}{|V|-1}$$

which is the highest possible value for density in G_c'. This is because no matter what mass you subtract from the previous mass, the denominator will

be the same, $|V|-1$. This corresponds to the case when there is no u specific edge that gets removed when v having $d_w(v)_{min}$ is removed. This would be the best case. This proves the left side of the lemma, $\frac{mass(G_c)-d_w(v)_{min}}{|V|-1} \geq density(G'_c)$.

Else if $\frac{d_u(v)_{min}}{d(v)_{min}} > 0$, and let's suppose that $\frac{d_u(v)_{min}}{d(v)_{min}} = \alpha, \alpha \in (0,1]$, then *USNAP* may pick any $b \in V(G_c)$ such that

$$d_w(b)(1+\beta) \leq d_w(v)_{min}(1+\alpha) where\, \alpha, \in (0,1] and \beta \in [0,1]$$

and we know that $d_w(v)_{min} < d_w(b)$ by definition. Thus, we have

$$d_w(b) \leq d_w(v)_{min}\frac{(1+\alpha)}{(1+\beta)}$$

To get an upper bound for $d_w(b)$, α would be 1 and β would be 0. Therefore,

$$d_w(b) \leq d_w(v)_{min}\frac{(1+\alpha)}{(1+\beta)} \leq 2d_w(v)_{min}$$

In this case, the removal of b will result in

$$density(G'_c) \geq \frac{mass(G_c) - 2d_w(v)_{min}}{|V|-1},$$

with the worst case being

$$density(G'_c) = \frac{mass(G_c) - 2d_w(v)_{min}}{|V|-1}$$

This proves the right hand side of the lemma, $density(G'_c) \geq \frac{mass(G_c)-2d_w(v)_{min}}{|V|-1}$. □

Lemma 1 proves that in each step of the greedy algorithm, $density(G'_c)$ would not be worse than $\frac{mass(G_c)-2d_w(v)_{min}}{|V|-1}$. The function that is used to select which v to remove in $V(G_c)$ in lemma 1 has two aspects: $d_w(v)$ is to have dense subgraphs, and $(1+\frac{d_u(v)}{d(v)})$ is to discourage the removal of nodes that have u specific edge(s). The more u specific edges v has, the greater $(1+\frac{d_u(v)}{d(v)})$ will be. 1 is added to $\frac{d_u(v)}{d(v)}$ in order to prevent the return of a tie score, 0, if $i, j \in V(G_c)$, $d_w(i) > d_w(j)$, and both don't have any u specific edge. This is because if the function is $(d_w(v)\frac{d_u(v)}{d(v)})$, then when $d_u = 0$, the score will be 0. This would be problematic if $d_w(i) >> d_w(j)$ and *USNAP* removes i instead of j since they both have a score of 0.

Lemma 2. *USNAP has a time complexity of $O(m + m_c logn_c + n_c logn_c)$.*

Proof. *USNAP* creates a collapsed graph from the input set of T snapshot graphs which takes $O(m)$. In CreateCollapseGraph($\mathcal{G}, u$), the loop in lines $2-7$ executes m times. The loop in lines $9-13$ executes m_c times as $|E(G_u)| =$

$|E(G_c)|$, and compute weights in line 12 takes constant time. Thus, *USNAP*'s line 2 takes $O(m)$.

The call to FindARegion(G_c, γ) in line 4 takes $O(m_c logn_c + n_c logn_c)$, and FindARegion$(G_c, \gamma)$ is the bottleneck in the while loop for *USNAP*.

In FindARegion(G_c, γ), line 2 takes $O(n_c)$ to initialize MH. Lines $5, 6, 9$ take constant time as variables are updated, and are not computed from scratch. The bottleneck for FindARegion(G_c, γ) is in line 4, RemoveNode(G_c, MH).

Line 1 of RemoveNode(G_c, MH) takes $O(logn_c)$ as it performs the delete minimum operation in MH. Lines $1, 2$ of RemoveNode(G_c, MH) execute n_c times as each vertex is to be removed once. Thus, lines $1-2$ take $O(n_c logn_c)$. Lines $3-5$ in RemoveNode(G_c, MH) execute m_c times as each edge is to be updated once, and line 4 takes $O(logn_c)$ as it performs the decrease key operation in MH. Thus, lines $3-5$ take $O(m_c logn_c)$. Therefore, the time complexity for FindARegion(G_c, γ) is $O(m_c logn_c + n_c logn_c)$.

In *USNAP*, FindARegion(G_c, γ) is executed at most $k + k'$ times, where both k, k' are constants. Therefore, the time complexity of *USNAP* is $O(m + m_c logn_c + n_c logn_c)$.

Since $m_c \leq m$ and $n_c \leq n$, the time complexity of *USNAP* in terms of the number of edges and number of vertices in the input set of T snapshot graphs is $O(mlogn + nlogn)$. Expressing the time complexity with m_c, n_c provides a tighter bound for *USNAP*.

□

Theorem 1. The removal of a vertex in each iteration according to Lemma 1 gives a 4 approximation for $density(G_c)$.

Proof. We obtain Theorem 1 by putting Lemma 3 and 4 together. □

This proof is adopted from Charikar's proof for his greedy approximation algorithm (Charikar [2000]). We first give an upper bound for the optimal solution. We assign each weighted edge $i\,j \in E(G_c)$ to either vertex i or j. For each vertex $i \in V(G_c)$, $d_a(i)$ denotes the sum of the edge weights for those edges that are assigned to i. Let $d_a^{max} = \underset{i \in V(G_c)}{max}\, d_a(i)$.

Lemma 3.

$$\underset{S_c \subseteq V}{max}\ \{density(S_c)\} \leq d_a^{max}\ for\ any\ edge\ assignment.$$

Proof. Consider S_c which maximizes density(S_c). Each edge in $E(S_c)$ is assigned to one vertex in S_c.

$$mass(S_c) \leq |S_c| \cdot d_a^{max}$$

$$\frac{mass(S_c)}{|S_c|} \leq d_a^{max}$$

$$density(S_c) \leq d_a^{max}$$

□

Now, let's consider the edge assignment for the greedy algorithm. All edges are unassigned initially. When the vertex with the minimum value according to Lemma 1 is deleted from S_c, the vertex is assigned all edges that connect from this vertex to the rest of vertices in S_c. All edges between any 2 vertices in the current set S_c are unassigned, and the rest of the edges are assigned. All edges are assigned at the end of the execution of the greedy algorithm.

Lemma 4. *Let α be the maximum value of density(S_c) over all sets of S_c obtained from removing one vertex at a time in each iteration according to Lemma 1. Then $d_a^{max} \leq 4\alpha$.*

Proof. Let's consider a single iteration. Let i_{min} be the vertex that is to be deleted, and i_{min} is selected according to lemma 1, i.e. i_{min} has the minimum value on $d_w(v)\,(1+\frac{du(v)}{d(v)})$ for all $v \in V(S_c)$ $(lemma\,1)$. From lemma 1, we know that $d_w(v)_{min} \leq d_w(i_{min}) \leq 2d_w(v)_{min}$. Recall $d_w(v)_{min}$ denote the minimum of $d_w(v)\,\forall v \in V(G_c)$. Note also that $\frac{2mass(S_c)}{|S_c|}$ is the average weighted degree, and thus $d_w(v)_{min} \leq \frac{2mass(S_c)}{|S_c|}$. Therefore,
$d_a(i_{min}) \leq d_w(i_{min}) \leq 2d_w(v)_{min} \leq \frac{4mass(S_c)}{|S_c|} \leq 4\alpha$. $d_a^{max} = \max_{i \in V(G_c)} d_a(i)$ i.e., over all iterations. Therefore $d_a^{max} \leq 4\alpha$.

□

Putting together Lemma 3 and Lemma 4, we obtained Theorem 1. We designed the removal of vertex as in Lemma 1 in order to find not only dense subgraphs, but unique dense subgraphs. The bound in Theorem 1 has not accounted for satisfying the exclusive threshold. However, from the validation of *USNAP* on real world datasets, we have shown that *USNAP* works well in practice.

2 Input

2.1 Datasets and data sources

4 NSCLC microarray gene expression datasets (Chitale et al. [2009], Okayama et al. [2012], Raponi et al. [2006]) obtained from the Gene Expression Omnibus database (Edgar et al. [2002]), and the Memorial Sloan-Kettering Cancer Center were used to demonstrate the effectiveness of *USNAP*. The number of stages, the number of samples, and the platform used were criteria for dataset selection. In this paper, we refer to the 4 datasets as *ChitaleMA1*, *ChitaleMA2* (both were from `http://cbio.mskcc.org/public/lung_array_data/`), *Okayama* (GSE31210) and *Raponi* (GSE4573). Protein-protein interaction data used was from Integrated Interactions Database (IID) version 2018-11 (Kotlyar et al. [2019]). Refer to Table 1 for more information regarding the data used.

Table 1: Input dynamic graphs to *USNAP*

Dataset	Histology	Stage	Nodes	Edges
ChitaleMA1	Adenocarcinoma	1A	5632	23314
ChitaleMA1	Adenocarcinoma	1B	4796	19127
ChitaleMA1	Adenocarcinoma	2B	6054	14843
ChitaleMA1	Adenocarcinoma	3A	5771	18071
ChitaleMA2	Adenocarcinoma	IA	6245	28431
ChitaleMA2	Adenocarcinoma	IB	6391	24172
ChitaleMA2	Adenocarcinoma	IIIA	5945	17641
Okayama	Adenocarcinoma	IA	7690	46697
Okayama	Adenocarcinoma	IB	7169	44028
Okayama	Adenocarcinoma	II	8157	41954
Raponi	Squamous cell carcinoma	Ia	5656	18881
Raponi	Squamous cell carcinoma	Ib	6192	23117
Raponi	Squamous cell carcinoma	IIb	6027	22705
Raponi	Squamous cell carcinoma	IIIa	5931	17413

2.2 Construction of the dynamic graphs

We generated the dynamic graphs for each dataset using the following approach, for each stage in each dataset:

- pairwise Pearson correlations were calculated for all gene pairs
- edges were ranked based on their absolute correlation values
- gene pairs with the top 1% of the absolute values were selected
- the selected correlated gene pairs were only considered if they had an overlap with protein-protein interactions.

3 Results

Tables $2-5$ in the supplementary material show the top 10 (or less if there were fewer results) densest *usnaps* having more than 2 nodes that are specific to stage 1A, stage 1B, stage 2 or 2B and stage 3A for each dataset respectively. The tables are in sorted order of density with $d0$ being the densest *usnap* for each stage and for each dataset. There could be ties for the $10th$ place but only one of them is included. All results are available at `https://www.cs.utoronto.ca/~juris/data/USNAP22`.

#Edge in 2/2B is used as a column heading because *ChitaleMA1* and *Raponi* have stage 2B data, and *Okayama* has stage 2 data. NAs do not mean that *USNAP* is unable to return results, but rather, NAs indicate that a given dataset

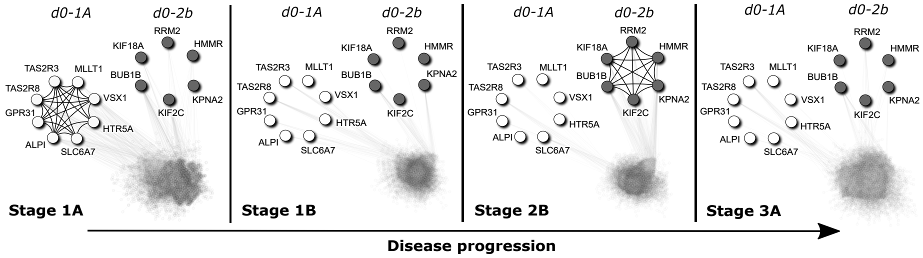

Figure 1: *USNAP* detects unique dense subgraphs in the Raponi dataset. *Usnap* $d0$ subgraph specific to stage 1A ($d0 - 1A$) is shown as open circles, and *usnap* $d0$ subgraph specific to stage 2B ($d0-2B$) is shown as closed circles. $d0-1A$ is only present in 1A, and not in any other stages; $d0 - 2B$ is only present in 2B, and not in any other stages (see the corresponding $d0 - 1A$, $d0 - 2B$ in other disease stages.) $d0-1A$ is composed of genes implicated in asthma, and asthma has been implicated as a risk factor for lung cancer.

does not contain a particular stage. For example, the dynamic graph that represents *ChitaleMA2* does not have stage 2 or 2B (refer to Table 1), and thus, NAs are placed in the #Edge in 2/2B columns for *ChitaleMA2*.

Importantly, for all *usnaps* from all 4 datasets, the edges that are specific to a stage do not appear in any other stages in the same dataset. For example, *usnap* $d0$ in *Raponi* has a clique with 15 edges that are specific to stage 2B (depicted in closed circles in Fig.1). Out of these 15 edges, no edge is present in stage 1A, 1B or 3A of *Raponi*.

Moreover, *USNAP* returns *usnaps* that are specific induced subgraphs for a given stage in the dataset; that is, all edges among the subgraph's nodes in the specific stage will be returned. For example, *usnap* $d1$ that is specific to stage 1A in *chitaleMA1* returns 18 edges among 7 nodes. This means that in stage 1A of *chitaleMA1*, there are only these 18 edges among these 7 nodes, and stage 1B, 2B and 3A in *chitaleMA1* do not have any of these 18 edges among these 7 nodes.

Table 2: *USNAP*'s results specific to stage 1A

Dataset	Id	Density	#Node in 1A	#Edge in 1A	#Edge in 1B	#Edge in 2/2B	#Edge in 3A
ChitaleMA1	d0	20.81	11	48	0	0	0
ChitaleMA1	d1	12.27	7	18	0	0	0
ChitaleMA1	d2	11.93	6	15	0	0	0
ChitaleMA1	d3	11.58	14	34	0	0	0
ChitaleMA1	d4	11.37	13	31	0	0	0
ChitaleMA1	d5	11.13	6	14	0	0	0
ChitaleMA1	d6	11.13	6	14	0	0	0
ChitaleMA1	d7	10.90	7	16	0	0	0
ChitaleMA1	d8	10.34	6	13	0	0	0
ChitaleMA1	d9	9.54	5	10	0	0	0
chitaleMA2	d0	18.20	12	52	0	NA	0
chitaleMA2	d1	14.17	8	27	0	NA	0
chitaleMA2	d2	13.12	8	25	0	NA	0
chitaleMA2	d3	12.00	7	20	0	NA	0
chitaleMA2	d4	11.40	7	19	0	NA	0
chitaleMA2	d5	10.20	7	17	0	NA	0
chitaleMA2	d6	9.10	6	13	0	NA	0
chitaleMA2	d7	9.10	6	13	0	NA	0
chitaleMA2	d8	8.40	5	10	0	NA	0
chitaleMA2	d9	7.70	6	11	0	NA	0
Okayama	d0	8.4	5	10	0	0	NA
Okayama	d1	5.25	4	5	0	0	NA
Okayama	d2	4.2	3	3	0	0	NA
Okayama	d3	2.8	3	2	0	0	NA
Okayama	d4	2.8	3	2	0	0	NA
Okayama	d5	2.8	3	2	0	0	NA
Okayama	d6	2.8	3	2	0	0	NA
Okayama	d7	2.8	3	2	0	0	NA
Raponi	d0	14.31	8	24	0	0	0
Raponi	d1	13.63	7	20	0	0	0
Raponi	d2	11.58	7	17	0	0	0
Raponi	d3	11.13	6	14	0	0	0
Raponi	d4	10.90	7	16	0	0	0
Raponi	d5	10.73	8	18	0	0	0
Raponi	d6	10.68	21	47	0	0	0
Raponi	d7	10.22	7	15	0	0	0
Raponi	d8	9.54	5	10	0	0	0
Raponi	d9	9.54	6	12	0	0	0

Table 3: *USNAP*'s results specific to stage 1B

Dataset	Id	Density	#Node in 1B	#Edge in 1B	#Edge in 1A	#Edge in 2/2B	#Edge in 3A
ChitaleMA1	d0	16.96	9	32	0	0	0
ChitaleMA1	d1	14.31	8	24	0	0	0
ChitaleMA1	d2	11.93	6	15	0	0	0
ChitaleMA1	d3	11.93	6	15	0	0	0
ChitaleMA1	d4	10.90	7	16	0	0	0
ChitaleMA1	d5	10.07	9	19	0	0	0
ChitaleMA1	d6	9.54	6	12	0	0	0
ChitaleMA1	d7	9.54	5	10	0	0	0
ChitaleMA1	d8	8.75	6	11	0	0	0
ChitaleMA1	d9	8.59	5	9	0	0	0
ChitaleMA2	d0	10.20	7	17	0	NA	0
ChitaleMA2	d1	6.30	4	6	0	NA	0
ChitaleMA2	d2	5.25	4	5	0	NA	0
ChitaleMA2	d3	5.25	4	5	0	NA	0
ChitaleMA2	d4	4.20	3	3	0	NA	0
ChitaleMA2	d5	4.20	3	3	0	NA	0
ChitaleMA2	d6	4.20	3	3	0	NA	0
ChitaleMA2	d7	4.20	3	3	0	NA	0
ChitaleMA2	d8	4.20	3	3	0	NA	0
ChitaleMA2	d9	2.80	3	2	0	NA	0
Okayama	d0	7.7	6	11	0	0	NA
Okayama	d1	6.72	5	8	0	0	NA
Okayama	d2	5.25	4	5	0	0	NA
Okayama	d3	5.25	4	5	0	0	NA
Okayama	d4	5.25	4	5	0	0	NA
Okayama	d5	4.2	3	3	0	0	NA
Okayama	d6	4.2	4	4	0	0	NA
Okayama	d7	4.2	3	3	0	0	NA
Okayama	d8	4.2	3	3	0	0	NA
Okayama	d9	4.2	4	4	0	0	NA
Raponi	d0	12.72	18	48	0	0	0
Raponi	d1	10.34	6	13	0	0	0
Raponi	d2	9.54	5	10	0	0	0
Raponi	d3	8.75	6	11	0	0	0
Raponi	d4	8.75	6	11	0	0	0
Raponi	d5	8.59	5	9	0	0	0
Raponi	d6	8.59	5	9	0	0	0
Raponi	d7	8.59	5	9	0	0	0
Raponi	d8	7.15	4	6	0	0	0
Raponi	d9	7.15	4	6	0	0	0

Table 4: *USNAP*'s results specific to stage 2 or 2B

Dataset	Id	Density	#Node in 2/2B	#Edge in 2/2B	#Edge in 1A	#Edge in 1B	#Edge in 3A
ChitaleMA1	d0	15.90	12	40	0	0	0
ChitaleMA1	d1	12.52	8	21	0	0	0
ChitaleMA1	d2	11.66	9	22	0	0	0
ChitaleMA1	d3	11.13	9	21	0	0	0
ChitaleMA1	d4	10.60	9	20	0	0	0
ChitaleMA1	d5	10.02	10	21	0	0	0
ChitaleMA1	d6	9.54	5	10	0	0	0
ChitaleMA1	d7	9.01	18	34	0	0	0
ChitaleMA1	d8	8.67	11	20	0	0	0
ChitaleMA1	d9	8.59	5	9	0	0	0
Okayama	d0	29.40	15	105	0	0	NA
Okayama	d1	8.40	5	10	0	0	NA
Okayama	d2	4.20	3	3	0	0	NA
Raponi	d0	11.93	6	15	0	0	0
Raponi	d1	11.93	8	20	0	0	0
Raponi	d2	11.13	6	14	0	0	0
Raponi	d3	10.34	6	13	0	0	0
Raponi	d4	9.54	6	12	0	0	0
Raponi	d5	9.54	6	12	0	0	0
Raponi	d6	9.54	5	10	0	0	0
Raponi	d7	9.54	6	12	0	0	0
Raponi	d8	8.94	8	15	0	0	0
Raponi	d9	8.94	8	15	0	0	0

Table 5: *USNAP*'s results specific to stage 3A

Dataset	Id	Density	#Node in 3A	#Edge in 3A	#Edge in 1A	#Edge in 1B	#Edge in 2/2B
ChitaleMa1	d0	13.71	8	23	0	0	0
ChitaleMa1	d1	12.27	7	18	0	0	0
ChitaleMa1	d2	11.93	8	20	0	0	0
ChitaleMa1	d3	11.58	7	17	0	0	0
ChitaleMa1	d4	11.58	7	17	0	0	0
ChitaleMa1	d5	11.13	9	21	0	0	0
ChitaleMa1	d6	11.13	18	42	0	0	0
ChitaleMa1	d7	10.90	7	16	0	0	0
ChitaleMa1	d8	10.84	11	25	0	0	0
ChitaleMa1	d9	10.22	7	15	0	0	0
ChitaleMA2	d0	12.00	7	20	0	0	NA
ChitaleMA2	d1	9.80	6	14	0	0	NA
ChitaleMA2	d2	9.10	6	13	0	0	NA
ChitaleMA2	d3	8.87	9	19	0	0	NA
ChitaleMA2	d4	8.40	6	12	0	0	NA
ChitaleMA2	d5	8.40	5	10	0	0	NA
ChitaleMA2	d6	7.20	7	12	0	0	NA
ChitaleMA2	d7	7.00	6	10	0	0	NA
ChitaleMA2	d8	6.72	5	8	0	0	NA
ChitaleMA2	d9	5.88	5	7	0	0	NA
Raponi	d0	11.13	6	14	0	0	0
Raponi	d1	11.13	6	14	0	0	0
Raponi	d2	11.13	6	14	0	0	0
Raponi	d3	10.73	8	18	0	0	0
Raponi	d4	9.54	7	14	0	0	0
Raponi	d5	9.54	5	10	0	0	0
Raponi	d6	9.30	20	39	0	0	0
Raponi	d7	8.75	6	11	0	0	0
Raponi	d8	7.15	4	6	0	0	0
Raponi	d9	7.15	4	6	0	0	0

Table 6: Pathway specific to stage 1A

Pathway	P-value (largest among the datasets)	No. Dataset
REACTOME-apoptosis	0.00202	2
PID-validated nuclear estrogen receptor alpha network	0.03936	2
BioCarta-the prc2 complex sets long-term gene silencing through modification of histone tails	0.00421	2
WikiPathways-fas ligand (fasl) pathway and stress induction of heat shock proteins (hsp) regulation	0.04963	2
RB-Pathways-apoptosis-entry-module	0.02770	2
WikiPathways-fluoropyrimidine activity	0.02161	2
WikiPathways-the effect of progerin on the involved genes in hutchinson-gilford progeria syndrome	0.00658	2
BioCarta-cyclin e destruction	0.02206	2
BioCarta-e2f1 destruction	0.02945	2
WikiPathways-dna mismatch repair	0.00139	2
BioCarta-caspase cascade in apoptosis	0.01883	2
REACTOME-mitotic metaphase/anaphase transition	0.01432	2

Table 7: Pathway specific to stage 1B

Pathway	P-value (largest among the datasets)	No. Dataset
REACTOME-interactions of rev with host cellular proteins	0.01320	2
REACTOME-early phase of hiv life cycle	0.04907	2
BioCarta-hiv-1 defeats host-mediated resistance by cem15	0.00820	2

Table 8: Pathway specific to stage 2or2B

Pathway	P-value (largest among the datasets)	No. Dataset
REACTOME-regulation of actin dynamics for phagocytic cup formation	0.04444	2
REACTOME-translocation of zap-70 to immunological synapse	0.00510	2
IPAVS-ang-ii-and-jak-stat-interactions-in-mediating-cardiac-myocyte-function	0.02776	2
ACSN2-cytoskeleton-polarity	0.03018	2
ACSN2-tcr-signaling	0.04455	2
REACTOME-interleukin-12 family signaling	0.03793	2
ACSN2-death-receptor	0.04746	2
KEGG-b cell receptor signaling	0.00239	2
KEGG-th1 and th2 cell differentiation	0.00005	2
IPAVS-gp130-jak-stat	0.00004	2
REACTOME-interleukin-2 family signaling	0.01162	2
REACTOME-negative regulation of the pi3k/akt network	0.00168	2
REACTOME-pi5p, pp2a and ier3 regulate pi3k/akt signaling	0.00104	2
REACTOME-signaling by erythropoietin	0.03272	2
KEGG-shigellosis	0.04444	2
stke-interleukin 4 (il-4) pathway	0.01749	2
PID-il5-mediated signaling events	0.02349	2
NetPath-bdnf	0.02386	2
REACTOME-regulation of kit signaling	0.00127	2
PID-epo signaling	0.00441	2
PID-il23-mediated signaling events	0.00554	2
KEGG-intestinal immune network for iga production	0.01930	2
REACTOME-nucleotide-binding domain, leucine rich repeat containing receptor (nlr) signaling	0.02938	2
PID-role of calcineurin-dependent nfat signaling in lymphocytes	0.03015	2
PID-a6b1 and a6b4 integrin signaling	0.01394	2
INOH-cd4 t cell receptor signaling-erk cascade	0.00008	2
PID-vegfr3 signaling in lymphatic endothelium	0.01138	2
BioCarta-growth hormone signaling	0.00857	2
REACTOME-erythropoietin activates ras	0.00719	2
REACTOME-interleukin-20 family signaling	0.04363	2
REACTOME-interleukin-35 signalling	0.02448	2
REACTOME-mapk3 (erk1) activation	0.01138	2
WikiPathways-t-cell receptor and co-stimulatory signaling	0.00014	2

Table 9: Pathway specific to stage 3A

Pathway	P-value (largest among the datasets)	No. Dataset
WikiPathways-parkin-ubiquitin proteasomal system	0.01909	2
WikiPathways-electron transport chain (oxphos system in mitochondria)	0.04956	2

References

Moses Charikar. Greedy approximation algorithms for finding dense components in a graph. In *International Workshop on Approximation Algorithms for Combinatorial Optimization*, pages 84–95. Springer, 2000.

D Chitale, Y Gong, B S Taylor, S Broderick, C Brennan, R Somwar, B Golas, L Wang, N Motoi, J Szoke, J M Reinersman, J Major, C Sander, V E Seshan, M F Zakowski, V Rusch, W Pao, W Gerald, and M Ladanyi. An integrated genomic analysis of lung cancer reveals loss of DUSP4 in EGFR-mutant tumors. *Oncogene*, 28(31):2773–83, 8 2009. ISSN 1476-5594. doi: 10.1038/onc.2009.135. URL http://www.nature.com/doifinder/10.1038/onc.2009.135http://www.ncbi.nlm.nih.gov/pubmed/19525976http://www.pubmedcentral.nih.gov/articlerender.fcgi?artid=PMC2722688.

R Edgar, M Domrachev, and A E Lash. Gene Expression Omnibus: NCBI gene expression and hybridization array data repository. *Nucleic Acids Res*, 30(1): 207–210, 1 2002.

Max Kotlyar, Chiara Pastrello, Zara Malik, and Igor Jurisica. IID 2018 update: context-specific physical protein-protein interactions in human, model organisms and domesticated species. *Nucleic Acids Res.*, 47(Database-Issue): D581–D589, 2019. doi: 10.1093/nar/gky1037. URL https://doi.org/10.1093/nar/gky1037.

H Okayama, T Kohno, Y Ishii, Y Shimada, K Shiraishi, R Iwakawa, K Furuta, K Tsuta, T Shibata, S Yamamoto, S Watanabe, H Sakamoto, K Kumamoto, S Takenoshita, N Gotoh, H Mizuno, A Sarai, S Kawano, R Yamaguchi, S Miyano, and J Yokota. Identification of genes upregulated in ALK-positive and EGFR/KRAS/ALK-negative lung adenocarcinomas. *Cancer Res*, 72(1): 100–111, 1 2012.

M Raponi, Y Zhang, J Yu, G Chen, G Lee, J M Taylor, J Macdonald, D Thomas, C Moskaluk, Y Wang, and D G Beer. Gene expression signatures for predicting prognosis of squamous cell and adenocarcinomas of the lung. *Cancer Res.*, 66(15):7466–7472, 8 2006.
